# Observations from behind the bar: changing patrons' behaviours in response to smoke-free legislation in Scotland

**DOI:** 10.1186/1471-2458-8-238

**Published:** 2008-07-14

**Authors:** Shona Hilton, Jane Cameron, Alice MacLean, Mark Petticrew

**Affiliations:** 1 MRC Social and Public Health Sciences Unit, Glasgow, UK; 2 Department of Environmental & Occupational Medicine, University of Aberdeen, Aberdeen, UK; 3 Public and Environmental Health research Unit, London School of Hygiene and Tropical Medicine, WC1E 7HT, UK

## Abstract

**Background:**

"Smoke-Free" legislation prohibiting smoking in all enclosed public places was introduced in March 2006. This qualitative study presents insights from bar workers about their observations of the changing social bar environment, changing patrons' behaviours and challenges bar workers have faced in managing smoke-free legislation.

**Methods:**

Twelve in-depth interviews were conducted between November 2006 and January 2007 with a purposively-selected sample of bar workers, identified from a larger quantitative study evaluating the impact of the legislation in Scotland [the Bar Workers' Health and Environmental Tobacco Smoke Exposure project (BHETSE)].

**Results:**

Bar workers all spoke of the improvements the legislation had brought to their working lives and the greater comfort it appeared to offer patrons. Bar workers reported that patrons were generally quick to accept and comply with the new law, and that families had become a greater feature of pub life since the legislation. However, they expressed concerns that older men seemed to have had most difficulty adjusting to the legislation and lack of knowledge about the best practices they should adopt in order to reduce the risks of unattended drinks being spiked and of anti-social behaviour associated with patrons moving outside to smoke.

**Conclusion:**

Smoke-free legislation is changing the social context of smoking in Scotland. Further research to assess the impact the legislation is having on older male smokers and on the incidence of drink spiking would be useful. More specifically, bar workers would benefit from guidance on how to manage issues arising from patrons moving outside to smoke.

## Background

Increasing evidence of the health risks posed by second-hand smoke (SHS) exposure [1-7] has been followed in recent years by the enactment of legislation designed to ensure workplaces and other public spaces are smoke-free. Following the example of other countries, such legislation was introduced in Scotland in March 2006 [8]. Its implementation was primarily a health and safety measure aimed at reducing the harmful effects of SHS to workers, particularly those in the hospitality sector who have been shown to be exposed to high levels of environmental tobacco smoke (ETS) in their workplace [5,8-11].

In countries where policies to restrict or prohibit smoking have been implemented, hospitality workers have been shown to benefit, particularly female bar workers from low socioecomonic status [12]. Bar workers respiratory function has been shown to improve [13] and there has been a positive shift in attitudes among bar workers towards preferring to work in a smoke-free environment [14]. Smoke-free legislation is also contributing towards wider public health gains as smoking becomes less socially acceptable [15,16] creating a social context which paves the way for increased smoking cessation [17], reduced consumption [16,18] and reduced initiation of smoking among the young [19,20]. Studies which report on how smoking behaviours have changed since smoking restrictions suggest that smoke-free public places may reduce smoking among young people [20] and support smoking cessation particularly in social venues such as pubs where there are increased cues to smoke [21] and where relapse is common [22,23].

Despite the strong public health justification for smoke-free legislation in Scotland and a high profile public awareness campaign about the dangers of SHS, concerns were voiced prior to its introduction about its potential negative economic impact and the difficulties of enforcing it. Whilst in the short-term there is some supportive evidence of reduced sales, lower customer numbers [24] and less support in deprived areas [25], compliance rates have in fact been consistently high as patrons have quickly adapted to the new law [26].

As yet however little has been written about how patrons' behaviours changed in response to the legislation [23], or how bar workers viewed and managed these changes in their bars. This study was therefore carried out to assess bar workers' reports of the changed environment, and in particular their perceptions of changes in patrons' behaviours as well as the challenges bar staff have faced in managing the smoke-free legislation.

## Methods

All twelve participants were identified and recruited from the Bar Workers' Health and Environmental Tobacco Smoke Exposure project (BHETSE) funded by NHS Health Scotland, which forms one part of a comprehensive evaluation programme of the smoke-free legislation in Scotland [27]. The BHETSE evaluation randomly recruited bars from a broad range of socio-economic areas and types of bars in urban, semi-urban and rural settings in and around Glasgow, Edinburgh and Aberdeen. The evaluation used convenience sampling to recruit bar workers to take part in three interviews over a year (in the months leading up to the ban, in the months after than ban and one year after the first interview).

To identify participants, BHETSE fieldworkers were asked to provide a list of names of bar workers willing to take part in a subsequent in-depth interview. The list of names (n = 26) was then cross-matched with the BHETSE database to draw a purposive sample of interviewees which ranged widely in terms of age, sex and work experience, as well as a range of socio-economic areas and settings (urban, semi-urban and rural). The final sampling frame was dictated by resources and included twelve bar workers, four from each city (Glasgow, Edinburgh and Aberdeen).

The interviews were conducted between November 2006 and January 2007 either in participants' own homes or workplaces or in a local academic department.

Each interview began with a general discussion about the smoking legislation and moved chronologically to enable participants to recount their observations and experiences from the period before, immediately after, and 8–10 months after the legislation was introduced. All the interviews were recorded with the participants' permission and extensive field notes made about each interview. Eleven interviews were transcribed fully verbatim and one interview was partly transcribed due to poor sound quality. Ethical approval for the study was obtained from the Faculty of Law, Business and Social Sciences Research Ethics Committee at the University of Glasgow.

Each transcript was repeatedly re-examined and cross-compared to identify common themes following the principle of the constant comparative method [28,29]. To enable further systematic comparisons to be made across data the framework approach to analysis was used to compare and link themes into a coding frame [30]. The use of the framework approach also highlighted deviant or contradictory data.

## Results

The twelve participants included seven men and five women aged between 24 and 67 years, with varying smoking status, levels of education, length of service in the hospitality sector, and number of hours they estimated they were exposed to SHS at work prior to the legislation (see table 1).

**Table 1:** The bars, bar workers and their work experience

**Bar worker**	**Sex**	**Age**	**Smoking status**	**Highest education level**	**Length service in hospitality sector (yrs)**	**Number of hrs ETS exposure per week at work**	**Bar location**	**Clientele**
GLA1	Male	33	Ex-smoker	Further Education	14	40	City Centre	Mixed clientele and clubbers
GLA2	Female	36	Regular smoker 20 cigarettes per day	School	3	18	City Centre	Mixed clientele, clubbers and passing trade
GLA3	Female	27	Regular smoker 15 cigarettes per day	Further Education	12	45	City Centre	Students, regular clientele after work and passing trade
GLA4	Male	34	Never smoked	University	13	35	Deprived residential area	Regulars and locals
ED1	Female	36	Never smoked	Further Education	4	50	City Centre	Mixed clientele and clubbers
ED2	Male	39	Regular smoker 15 cigars per day	School	19	105	Mixed residential area	Regulars and locals
ED3	Male	36	Never smoked	University	20	50	City Centre	Mixed clientele, clubbers and passing trade
ED4	Male	54	Regular smoker 10 cigars per day	University	26	42	Deprived central area	Regulars
ABN1	Male	51	Regular smoker 10 cigarettes per day	Further Education	6	56	Deprived central area	Regulars
ABN2	Male	24	Regular smoker 20 cigarettes per day	University	5	45	City centre	Students
ABN3	Female	67	Never smoked	School	20	70	Rural deprived area	Regulars and locals
ABN4	Female	43	Regular smoker 20 cigarettes per day	Further Education	10	7	Deprived central area	Regulars

Prior to the ban the main fear that bar workers expressed was about the impact the impeding ban would have on lost revenue and the potential for job losses. Post-ban, bar workers mentioned these concerns did not elaborate further, as they did not feel that their bars had been adversely affected. Before the ban two bar workers also mentioned concerns about how readily patrons would comply with the ban, but again when they were interviewed after the ban they did not focus on this issue because in their experience patrons had generally accepted the new legislation. During the interviews bar workers mainly chose to focus on their observations and experiences post-ban. Bar workers in this study generally supported the smoke-free legislation because they felt it had improved the air quality in bars, their health and because they felt there was general support for the legislation among patrons. Three main themes dominated the discussions after the ban: (1) changes in patrons' smoking behaviours (2) issues around the safety of patrons and (3) changes in bar social atmosphere. Each of these themes will be examined in turn.

### Changes in patrons' smoking behaviours

In relation to changes in patrons' smoking behaviours, bar workers stated that patrons readily accepted and complied with the new law and that following its introduction the main change was that smokers now had to move outside to smoke. It was common for bar workers to suggest that patrons were making the most of this situation by using it as an opportunity to develop new social networks. For example, one bar worker stated: "*People haven't minded going outside to smoke. In fact a lot of them are enjoying the company outside. Sort of meet a new circle of friends...*" (ED4: Male, aged 54 yrs, smoker). There was a sense among bar workers that the legislation had acted as a catalyst for these patrons who smoke to join together outside in a 'common purpose' and often with a sense of 'camaraderie'. One bar worker considered that it was: "*...like when you first started smoking at school round at the smokers' corner, it's like a sociable thing to do and where you went to speak to everybody, and it's just like that now*" (ABN4: Female, aged 43 yrs, smoker).

However, belying these positive social aspects of the changes were discourses about exclusion and some suggestion that smokers were now set apart from mainstream non-smoking society. For example, one bar worker, himself a smoker, referred to smokers as 'the unclean' (ED2: Male, aged 39 yrs, smoker) and bar workers often expressed their concerns that some older patrons who were smokers were excluded from being able to join the other smokers outside due to their frailty. For instance, one bar worker had noticed that she had not seen as many of her older patrons, "*...it's all like younger wans (standing outside), you know like sixty-under*" (GLA2: female, aged 36 yrs smoker). It was also common for bar workers to suggest that the older single men had been most negatively affected by the legislation and in some cases have stopped coming into the bar altogether. "*There's a few (older men) but they can't sit and have their cigarette, a lot of them stopped coming down. They're not wanting to go and stand in the cold*" (ABN4: Female, aged 43 yrs, smoker). Similarly, another bar worker recalled a conversation she had had recently with a regular patron:

"*...I was talking to an old guy the other night there and he was saying oh, well I can buy a can of Tennants for 60p out of Tesco's, so I would rather just sit at home and drink and smoke at home. I was, oh, well 'that's not very, it's not ideal is it?*"' (ED1: Female, aged 36 yrs, non smoker).

Although bar workers often made some sympathetic comments about the possible social isolation these men now face as a consequence of the new law, there was a general sense that the legislation was changing the social context of smoking and acceptance that it marked significant progress towards protecting bar staff and other patrons from the harmful effects of second-hand smoke.

### Concerns about patrons' safety

Another issue some bar workers expressed concern about was in relation to the safety of their patrons. This concern was two fold: 1) concern about an increase in drink fuelled street violence particularly at weekends and or after football matches and, 2) concern about an increased opportunity for the spiking of unattended drinks.

Since the legislation the increase in the number of patrons standing outside on the streets to smoke had increased the potential for street violence and posed challenges for bar staff trying to monitor drunken behaviour of patrons. One bar worker stated: "*... having you know, like a hundred and fifty smokers outside. It makes the door awfully difficult to watch*" (ABN3: female, aged 67 yrs, non smoker). Another bar worker explained that prior to the legislation his bar had a policy to lock its door during certain football matches to reduce the likelihood of his patrons (Rangers fans) clashing with the patrons (Celtic fans) of a neighbouring bar. He explained:

"*...since the smoking ban they've only played each other twice and the first game didn't matter much at all because the league was over then so it was fine, em... but the second game was obviously the start of the season so it was quite important, so you're talking at half-time mainly when everybody's just gonna pile outside, you're talking maybe sixty seventy guys from each place, so you've a hundred and forty guys standing outside you know almost next to each other and, you know, and if, if something happens, if there's a bad decision on the game or the league's deciding on it sort of thing, it could be a disaster, yeah.*" (GLA 4: Male, aged 34 yrs, non smoker)

Although some bar workers spoke about the difficulties in monitoring and managing patrons smoking since the legislation no bar workers spoke of any actual violent incidents.

The other concern about safety was in relation to patrons leaving drinks unattended thus increasing the opportunity for drinks to be spiked. Most bar workers spoke about this concern, particularly those workers employed in bars with no outdoor drinking area licence and those employed in city centre locations. For example, one bar worker in a large city centre bar spoke about the ways they have attempted to reduce the risk of spiking, stating that:

"*We're conscious of drinks getting spiked, etcetera, we haven't done anything apart from just customer awareness, you know, just keep an eye on your drink and that. And there is a table that they can put their drink just as they go out so, again with the windows you can see your drink all the time. You know, it's just trying to be proactive in that respect*"(GLA1: male, aged 33 yrs, Ex smoker).

However, some bar workers considered looking after people's drinks was impracticable and might lure people into a false sense of security. Bar workers were often unsure of the best advice to give and consequently the advice they gave varied. For example, one bar worker from a busy city centre bar stated: "*I always say to people if you're going away just give us a shout and I'll sit them either behind the bar or on the bar and be able to keep an eye on them*" (GLA3: female, aged 27 yrs, smoker). Whilst another bar worker advocated that patrons never leave their drinks unattended, stating: "*I encourage people to take their drinks with them, I mean this sort of like putting a bar mat on top of that's just saying, this drink is unattended*" (EH1: female, aged 36 yrs, non smoker). Bar worker were unsure whether placing beer mats over unattended drinks, or setting aside drinks tables for patrons to leave their drinks on while going outside to smoke are helpful strategies in reducing this risk, or merely serve to advertise the fact that drinks are unattended. Bar workers did not mention any specific incidents of drink spiking, but wanted to highlight the fact that they felt the smoking ban had increased any existing risk, and had also enhanced their sense that they were inadequately trained to deal with such incidents should they occur.

### Changes in bar social atmosphere

Bar workers spoke of the changes in bar atmosphere both from the perspective of bar workers place of work and as a place in which their patrons socialised. Most of the bar workers stated that they believed that since the legislation their working environment and health had improved and that they enjoyed working in a smoke-free environment. Even bar workers who smoked stated that they preferred working in a smoke-free environment. However, bar workers mentioned that smoke had been replaced by other unpleasant smells and that the relocation of smoking to doorways presented a poor image of the bar to passers-by, and more generally of the city to tourists. Indoors, bar workers considered that the legislation had led to improvements the overall appearance of the bar, and other aspects of the bar environment. Seven out of the 12 bars had undergone some level of redecoration. In the bars with few decorative improvements bar workers mentioned installing air fresheners, candles and increasing general ventilation in order to improve their working environment.

With regards to the social atmosphere of the bar, very few bar workers had noticed a negative change. One bar worker commented on the disruption of conversations between smokers and non-smokers when smokers would leave their company to smoke outside. Another bar worker spoke of patrons moving on more quickly: "*I mean it used to be people would come in and stay for two or three drinks, and now you get them in for one drink and they move, because in between that move they can have a cigarette*" (GLA2: female, aged 36 yrs, smoker).

However other bar workers felt that the bar atmosphere had improved since the legislation because they had observed an increase in the number of families and women as customers. For instance, one bar worker commented that their customer base has changed from mainly men to a mix of men, women and families. She also commented, "*... we have more trouble with people trying to bring their children into the pub. 'Cos we don't have a children's licence. But since the smoking ban, the perception is that because there's no smoking, you can bring your children*" (ED1: Female, aged 36 yrs, non smoker). Similarly, another bar worker stated:

"*I think you know, because we're doing more food, there's more families in, you hear more laughter and screaming, whereas before, if all your single guys are in, it's very quiet. Very much like a library*" (GLA1: male, aged 33, ex smoker).

All the interviewees spoke of the improvements that the smoke-free legislation had brought to their working lives and the greater comfort it provided for patrons. Bar workers seemed pleased with the relative ease with which their patrons had accepted and complied with the new law. Any residual concerns related to monitoring and managing the safety of their patrons, and in encouraging some of their older more vulnerable patrons to feel included in the social aspects of bar life.

## Discussion

The findings of this study are generally in accord with other studies, though as noted below several new issues relating to the effects of smoking restrictions were also identified. Firstly, bar workers in this study generally supported the smoke-free legislation because it was perceived to have improved the air quality in bars and health of bar workers [31,32] and, as in other studies, they, felt there was general support for the legislation since its implementation, from bar workers, patrons [33] and the public alike [34]. Similar to evaluations conducted elsewhere [26] our findings indicate that bar workers considered that patrons had readily accepted and complied with the new law and had even used the opportunity to develop new social networks. Indeed, this aspect of the legislation has attracted some media attention with the term 'smirting' coined to describe a new phenomenon of "smoking and flirting" [35,36].

To date little research has focused on the changing experience of public smoking. In this study, bar workers observed that the legislation had altered the social context of smoking by increasing the visibility of smokers in doorways and designated smoking areas outside the bar. This was deemed to be problematic as it might present a poor image both of the bar and city to passers-by. This resonates with the findings from evaluations of workplace smoking restrictions introducted by employers in the 1990s. In Scotland a phone survey of 200 Scottish employers, found that 26% of employers were experiencing problems with their restrictive smoking policy, including smoking in toilets and the negative corporate image portrayed when employees congregate outside the entrance to smoke [37]. This concern was also noted by Parry et al (2000) [38] who conducted a survey and interviews with staff at a Scottish university to evaluate smoking restrictions implemented within the university in 1997. Key findings that focused on the change in smoking behaviours were that whilst the ban was largely welcomed by staff key complaints were raised about non smokers having to run 'the smoking gauntlet' on their way in and from work as smokers congregated around doorway entrances to smoke. This resulted in ETS drifting back into the buildings, complaints about the increase in smoking debris around doorways which could constitute a fire hazard and concerns that smokers might portray a poor image of the university to outsiders. Whilst some staff expressed sympathy for their colleagues who had to smoke outside in harsh weather, the increased visibility of smoking also concerned them. This is one aspect of the legislation that needs further research in order to determine how young people will interpret such images of public smoking. Tobacco advertising has traditionally targeted the young with positive images of smoking in order to influence behaviour [39], while bans on advertising and successive health promotion campaigns have used negative images of smoking to reduce its appeal to young people [40]. On the one hand groups of smokers huddled together in door ways sheltering from inclement weather may do little to portray its attractiveness; on the other, the increased visibility of smokers in the context of obvious drinking and socialising may increase its appeal to some young people. This is an area requiring further research particularly among socially and economically marginalised communities where the prevalence of smoking is greatest, [41] support for the legislation lowest [25] and the stigmatisation of smokers possibly greatest [42]. Bar workers in this study viewed the greater numbers of children coming into bars as a positive consequence of the ban and there is support in the literature that encouraging a culture that accepts responsible social drinking as a normal part of life will have less alcohol abuse than cultures that fear and condemn alcohol [43]. As yet, it is not known whether children's early attendance in pubs will encourage them to into under-age drinking, although early initiation of drinking is associated with heavy drinking in adulthood [44].

Similar to reports of the effects of the smoke-free legislation in Ireland [45], this research highlighted bar workers' concerns that some older men faced particular difficulties in adapting to the legislation. In an Australian study Wakefield and colleagues have reported that older regular smokers felt that the ban in Melbourne, on smoking in restaurants, but not pubs, actually encouraged smoking, and that a ban in pubs would have assisted smokers to quit by reducing smoking cues and peer pressure to smoke [23]. It is perhaps unsurprising that older patrons with poor health and limited mobility may be most affected by having to stand outside in the poor Scottish weather. This does suggest that older smokers may require additional support to quit if smoking bans are not to affect them inequitably.

Bar workers in this study also expressed concern over spiking of drinks. There has been no mention of this problem in other post-legislation studies [13,17,33,46-48]. This may reflect the fact most evaluations have used survey methods less likely than in-depth interviews to pick up such an issue, or it may reflect an increased awareness about drink spiking in Scotland due to a campaign run by Strathclyde Police Force and media reports about the dangers of drink spiking [49-51]. Whilst there are no accurate statistics to indicate the extent of drink spiking in Scotland, The Roofie Foundation (an agency who operate in the area of drug-related rape and sexual abuse) report that in 2002, in England 935 drug rape incidents were reported. The majority of victims had their drinks spiked in a pub [52]. Although the risk overall is very low, and bar workers in this study did not mention witnessing any specific incidents of drink spiking, they felt the smoking ban had increased the existing risk and increased their concern that they were inadequately trained to deal with such an incident should it occur.

To our knowledge, this is the first published qualitative study to fully explore bar workers' observations of changes in Scottish bars since the introduction of the Smoke-Free Scotland legislation. Our findings must be interpreted with caution due to the small sample size, which was dictated by available resources. Nevertheless, this exploratory study offers original insights into bar workers' observations about the new legislation. We also anticipated that by examining these observations from the perspectives of bar workers, who are well placed to notice such changes, we would be able to identify issues that warrant further investigation and make recommendations that are of use to those seeking to implement legislation elsewhere. In the event there was considerable consistency in the views expressed and many of the themes which arose resonate with findings from other studies [33,34,45]. It is possible however that if we had conducted more interviews we would have identified more themes (or sub-themes).

The other main limitation relates to the relatively short length of follow-up. It is possible that the issues which the bar workers raised reflect a relatively short-term impact of the Smoke Free legislation, and any perceived effects may dissipate over a longer period of follow-up. How businesses and smokers accommodate (and may be helped to accommodate) to restrictions on smoking over longer periods may be a suitable subject for further research.

## Conclusion

This paper has presented bar workers' perceptions of changes in patrons' behaviours and in bar environments post-legislation as well as offering an insight into the challenges bar staff faced in managing the smoke-free legislation. These findings suggest that further research may usefully address the needs of older smokers and in particular whether smoking restrictions affect them differentially. Any new research also needs to include patrons from socially and economically marginalised communities where the prevalence of smoking is greatest, and support for the ban weakest. This research would usefully contribute to the development of support services for smoking cessation in older adults, and may help to guide health promotion campaigns to avoid the over-stigmatisation of smokers.

From this study it appeared that bar staff were concerned about the spiking of drinks, and about their lack of training to allow them to handle the problem. This suggests that training to assist bar workers to reduce the risk to patrons may be of value. This, and warning leaflets or posters, may be particularly important in venues where it is not possible for smokers to take their drinks outside. Information to increase awareness of spiking and how to avoid it is already available from NHS Direct [53].

It should be acknowledged however that the actual level of risk is difficult to ascertain, partly because drink spiking incidents largely go unreported. Recording these data as recommended by the Advisory Council on the Misuse Of Drugs-Drug Facilitated Sexual Abuse Report (2007) would be helpful in determining the true extent of the problem.

One final implication of this study is relevant to policymakers and others seeking to implement restrictions on smoking in clubs and bars; that is, that negative attitudes to the legislation may alter in the light of subsequent experience. In the current study the bar workers interviewed were supportive of the legislation, and spoke of the improvements the legislation had brought to their working lives and the greater comfort it appeared to offer patrons. They also noted that the physical and social aspects of bar environments improved. This was in contrast to the more negative expectations of bar workers before the ban.

This study presents original insights from bar workers about changes in patrons' behaviours and bar environments, and about the challenges bar workers have faced in managing smoke-free legislation in Scotland. The findings suggest that bar workers perceive real improvements in their working environment since the legislation was enacted, though they are concerned about their ability to deal with the risk of drink-spiking (though the size of the risk this poses is unclear). They also suggest that the smoking restrictions may differentially affect older smokers.

## Competing interests

We have no competing interests. This study was funded by the Medical Research Council and recruited bar workers from the BHETSE project funded by NHS Health Scotland and the Scottish Government, which forms one part of a comprehensive evaluation programme of the smoke-free legislation in Scotland. At the time of data collection Mark Petticrew was funded by the Chief Scientist Office of the Scottish Executive of Health.

## Authors' contributions

SH participated in the design, data collection, analysis and drafted the manuscript. MP commented on drafts of the manuscript and helped in drafting the manuscript. JC conducted the analysis. AM collected the data. All authors approved the final manuscript.

## Pre-publication history

The pre-publication history for this paper can be accessed here:


